# Fluid expansion improve ventriculo-arterial coupling in preload-dependent patients: a prospective observational study

**DOI:** 10.1186/s12871-020-01087-7

**Published:** 2020-07-17

**Authors:** Pierre Huette, Osama Abou-Arab, Dan Longrois, Pierre-Grégoire Guinot

**Affiliations:** 1 Anaesthesiology and Critical Care Department, Amiens University Hospital, Rond point Fernand Leger, F-80054 Amiens, France; 2 Anaesthesiology and Critical Care Department, Bichat Claude Bernard Hospital and INSERM1148, Paris, France; 3 Anaesthesiology and Critical Care Department, Dijon University Hospital, 2 Bd Maréchal de Lattre de Tassigny, F-21000 Dijon, France

**Keywords:** Ventricular-arterial coupling, Fluid therapy, Cardiac output, ICU

## Abstract

**Background:**

The objectives of the present study was to evaluate the effect of fluid challenge (FC) on ventriculo-arterial (V-A) coupling, its determinants: arterial elastance and ventricular elastance, and ability to predict fluid responsiveness.

**Methods:**

Thirty patients admitted to cardio-thoracic ICU in whom the physician decided to perform FC were included. Arterial pressure, cardiac output, arterial elastance, and ventricular elastance, were measured before and after FC with 500 ml of lactated Ringer’s solution. Fluid responders were defined as patients with more than a 15% increase in stroke volume. V-A coupling was evaluated by the arterial elastance to ventricular elastance ratio.

**Results:**

Twenty-three (77%) of the 30 patients included in the study were fluid responders. Before FC, responders had higher arterial elastance and arterial elastance to ventricular elastance ratio. FC significantly increased mean arterial pressure, stroke volume and cardiac output, and significantly decreased systemic vascular resistance, arterial elastance and consequently the arterial elastance to ventricular elastance ratio. Changes in arterial elastance were correlated with changes in stroke volume, systemic vascular resistance, and arterial compliance. Baseline arterial elastance to ventricular elastance ratio over 1.4 predicted fluid responsiveness (area under the curve [95% confidence interval]: 0.84 [0.66–1]; *p* < 0.0001).

**Conclusions:**

Fluid responsiveness patients had V-A coupling characterized by increase arterial elastance to ventricular elastance ratio, in relation to an increase arterial elastance. Fc improved the V-A coupling ratio by decreasing arterial elastance without altering ventricular elastance. Arterial elastance changes were related to those of systemic vascular resistance (continue component) and of arterial compliance (pulsatile component).

## Background

Fluid challenge (FC) is the most commonly performed bedside haemodynamic intervention in critical care medicine. In conventional haemodynamic analysis, cardiac output (CO) is considered to be a continuous function, and the heart and vascular system are considered separately. Two different concepts have therefore been developed [1, 2]. Guyton et al. considered the heart to be a pump driven by continuous flow from a purely resistive circuit - despite the pulsatile nature of this flow (mean atrial pressure – right atrial pressure = CO x systemic vascular resistance) [1]. Several authors subsequently developed a model of stroke volume (SV) based on the pressure-volume relationships of the ventricle and the vascular system [2–4]. This model considers left ventricular (LV) energetics, myocardial function and ventricular performance by taking into account the interaction between the ventricle and the vascular system. Hence, LV end-systolic elastance (E_LV_) corresponds to LV contractility and arterial elastance (E_A_) corresponds to the effective elastance of the arterial system [2–4]. Given that the ventricular and the arterial systems operate simultaneously, ventriculo-arterial (V-A) coupling (i.e the E_A_/E_LV_ ratio) reflects the mechanical efficiency of the cardiovascular system by analysing the interaction between cardiac performance and vascular function. V-A coupling determines the SV and the ejection pressure (i.e. arterial blood pressure) [2, 4].

Studies have demonstrated that V-A coupling can describe and characterize pathophysiological mechanisms, evaluate treatment effects, and may represent a parameter that describes the energetic cost [5–7]. Moreover, there is wide evidence that V-A coupling is a hemodynamic parameter that is associated with patient outcomes [5, 8, 9]. The relevance of V-A coupling as a parameter of hemodynamic optimization could be related to the fact that V-A coupling is a parameter of cardiovascular efficiency whereas the classical hemodynamic parameters are exclusively parameters of cardiovascular efficacy. The clinical relevance of this model is based on the fact that E_A_/E_LV_ predicts outcomes independently from other parameters [5, 8, 9].

The optimal hemodynamic intervention in patients with acute circulatory failure would improve efficacy with the lowest energetic cost (high efficiency) for the cardiovascular system (i.e lower E_A_/E_LV_ ratio) [8]. Because treatment of acute circulatory failure comprises several medications (fluid infusion, vasopressor use …), it would be of interest to know the effect of each treatment on V-A coupling [6, 8, 9]. Few studies have focused on FC from the perspective of the V-A coupling model [9]. A description of the cardiovascular effects of FC may improve our understanding of the pathophysiology of haemodynamic states.

The main objective of this study was therefore to evaluate the impact of FC on V-A coupling, and its determinants. The secondary objective was to determine the value of the pre-challenge E_A_/E_LV_ ratio as a predictor of a post-challenge increase in SV.

## Methods

### Ethics

The study’s objectives and procedures were approved by the local independent ethics committee (Comité de Protection des Personnes Nord-Ouest II, Amiens, France; RNI2014–39 on November 26th, 2014). According to French law, all patients received written information about the study and provided their verbal consent to participate. The present manuscript was drafted in compliance with the STROBE checklist for cohort studies [10].

### Patients

This prospective, observational study was performed in the Amiens University Hospital cardiothoracic ICU (Amiens, France) during 1 year between 2015 and 2016. The main inclusion criteria were as follows: age 18 or over, controlled positive ventilation, and a clinical decision to perform FC for volume expansion within the first hours of admission to ICU. Exclusion criteria were permanent arrhythmia, cardiac conduction block, pacemaker (or need for temporary pacemaker using epicardial wires), norepinephrine, epinephrine or dobutamine, poor echogenicity, aortic regurgitation, and right heart dysfunction. The indications for FC were arterial hypotension (systolic arterial pressure (SAP) less than 90 mmHg, or mean arterial pressure (MAP) less than 65 mmHg), or SV change greater than 10% during a passive leg raising manoeuvre, or clinical signs of hypoperfusion (skin mottling, and capillary refill time greater than 3 s).

### Haemodynamic parameters

Transthoracic echocardiography (with the CX50 ultrasound system and an S5–1 Sector Array Transducer, Philips Medical System, Suresnes, France) was performed by a physician blinded to the study outcomes. Left ventricular ejection fraction (LVEF), end-systolic volume (ESV), and end-diastolic volume (EDV) were measured using Simpson’s method on a four-chamber view. The aortic velocity-time integral (VTIAo), pre-ejection time and systolic time were measured by pulsed Doppler at the left ventricular outflow tract on a five-chamber view. Stroke volume (SV; mL) was calculated as VTIAo×SAo, and was expressed as indexed SV (SVi) = SV/body surface area (ml.m^− 2^). Cardiac output (CO) was calculated as SV × heart rate (HR), and was expressed as indexed CO (CI) = CO/ body surface area (ml min^− 1^ m^− 2^). Mean echocardiographic parameters were calculated from five measurements (regardless of the respiratory cycle) and analysed retrospectively.

### Left ventricular end-systolic elastance, arterial elastance, ventricular-arterial coupling

E_LV_, an index of ventricular contractility, was evaluated by using the non-invasive, single-beat method described by Chen et al. [11]. This method is based on the assumption that time-variation of left ventricular elastance is not influenced by loading conditions or heart rate. E_LV_ was calculated by the formula: E_LV_ = (Pd – (E_Nd(test)_ * Pes * 0.9)) / (SV * E_Nd(test)_). E_Nd(test)_ was obtained from a group-averaged normalized elastance curve value at this same time td (E_Nd(avg)_), baseline LVEF and the ratio of diastolic to systolic arterial pressure (Pd / Pes) [12]. E_Nd(avg)_ was determined by a seven-term polynomial function that includes the ratio of pre-ejection period to total systolic period [11]. We calculated the coefficient of variation (CV), precision and least significant change (LSC) for E_LV_ in the first ten patients. CV was 7.7% ± 0.6 and LSC was 10.9% ± 0.8.

Sunagawa et al. demonstrated that arterial load could be characterized in the time domain as arterial elastance (E_A_) [2]. E_A_ was calculated as end systolic pressure (ESP)/ SV (mmHg ml^− 1^) where ESP is 0.9 x systolic arterial pressure (SAP) [13].

Arterial pressure was measured by an invasive radial artery approach. In healthy men and women, mean E_A_/E_LV_, E_A_, and E_LV_ values measured invasively at rest are 1.0 ± 0.36, 2.2 ± 0.8 mmHg.ml^− 1^, and 2.3 ± 1.0 mmHg.ml^− 1^, respectively [14, 15]. In the present study, patients with an E_A_/E_LV_ ratio over 1.36 were classified as uncoupled [15].

The total energy generated by each cardiac contraction is called the “pressure-volume area” (PVA), corresponding to the sum of the external mechanical work exerted during systole (SW) and the potential energy (PE) stored at the end of systole: PVA = SW + PE [16]. SW is calculated as end systolic pressure (ESP) x SV. PE is calculated as ESP x ((ESV-V_0_)/2), and assumes that V_0_ is negligible compared to ESV. The SW/PVA ratio corresponds to the mechanical efficiency of converting the total mechanical energy (PVA) available to the left ventricular SW [16]. The SW/PVA ratio is associated to myocardial oxygen consumption.

Indexed systemic vascular resistance (SVRi) (mmHg.ml^-1.^m^− 2^) was calculated as MAP-central venous pressure (CVP)/ cardiac index (CI), and total arterial compliance (C_A_) (ml.mmHg^− 1^) was calculated as SV/arterial pulse pressure [17].

### Study procedures

The following clinical parameters were recorded: demographic, ventilation parameters, and primary diagnosis. After an equilibration period, capillary refill time (measured at the distal phalanx of the index finger), heart rate (HR), systolic arterial pressure (SAP), MAP, diastolic arterial pressure (DAP), CVP, SVi, CI, EDV, ESV, pre-ejection time, systolic time interval, and blood gas levels were measured at baseline. In the present study, FC always consisted of a 10-min infusion of 500 ml of lactated Ringer’s solution [18, 19]. A second set of measurements was performed immediately after FC. All patients were mechanically ventilated in volume-controlled mode with a tidal volume set at 7–9 ml kg^− 1^ ideal body weight, and a positive end-expiratory pressure (PEEP) of 5–8 cmH_2_O. Ventilator settings were not modified during the study period.

### Statistical analysis

The sample size was calculated on the reproducibility initially measured in the study reported by Chen et al. [11]. With a reproducibility of 20%, we calculated that a sample of thirty patients would be sufficient to demonstrate an absolute change of more than 20% in the E_A_/E_LV_ ratio in response to FC. The distribution of the variables was assessed by a Shapiro-Wilk test. Data are expressed as number, proportion (in per cent), mean ± standard deviation (SD) or median [interquartile range (IQR)], as appropriate. Fluid response was defined as a greater than 15% increase in SV after FC [19, 20]. This cutoff value was considered to be clinically relevant and in accordance with measurement variability. The non-parametric Wilcoxon rank sum test, Student’s paired t test, Student’s t test, and the Mann-Whitney test were used to assess statistical significance, as appropriate. A receiver-operating characteristic curve was established for the ability of E_A_, E_LV_, the E_A_/E_LV_ ratio to predict a greater than 15% increase in SV. The limit for statistical significance was *p* < 0.05. SPSS® software (version 22, IBM, New York, NY, USA) was used for all statistical analyses.

## Results

**Fig. 1:**
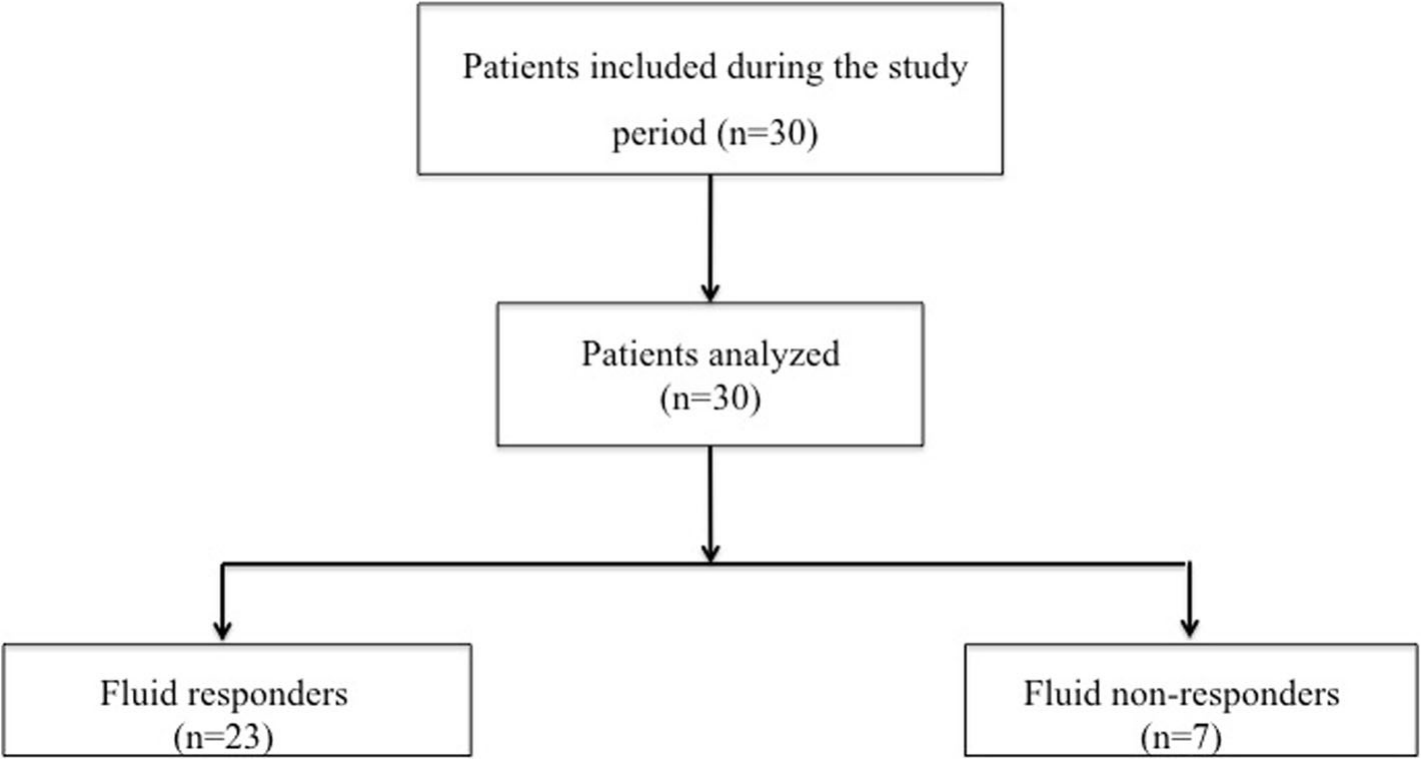
Study flow chart

Thirty patients were included and analysed (Fig. 1). These patients had undergone cardiovascular surgery (*n* = 29) or thoracic surgery (*n* = 1) (Table 1). The main indications for fluid expansion were arterial hypotension (*n* = 17), SV change greater than 10% with PLR (*n* = 9), skin mottling (*n* = 4). No significant difference in indications was observed between SV responders and SV non-responders (*p* = 0.336). Values for E_A_, E_LV_ and E_A_/E_LV_ ratio were not significantly different between men and women, or according to type of surgery or medical characteristics (*p*-value > 0.05), therefore allowing pooled analysis (Table 1). No patients developed complications (arrhythmia, hypoxaemia, left heart failure) during FC.

**Table 1:** Baseline characteristics of the study participants. Values are expressed as mean ± SD or number (%). CABG: coronary artery bypass graft. *P* value refers to comparison between ventriculo-arterial uncoupled and ventriculo-arterial coupled patients. Abnormal ventriculo-arterial coupling was defined as a E_A_/E_LV_ ratio greater than 1.36

Variables	Ventriculo-arterial coupled patients (***n*** = 7)	Ventriculo-arterial uncoupled patients (***n*** = 23)	***P*** value
Age (mean (SD), years)	66 (13)	66 (12)	0.947
Gender (F/M)	1/6	7/16	0.638
Disease, n (%)			
Arterial hypertension	5 (71)	14 (61)	1
Aortic stenosis	5 (71)	14 (61)	1
Diabetes mellitus	3 (43)	3 (13)	0.120
Dyslipidaemia	5 (71)	11 (48)	0.399
Smoking	4 (57)	10 (44)	0.675
Heart surgery, n (%)			
Valve replacement	5 (71)	12 (52)	
CABG	1 (14)	5 (22)	
Mixed	0	4 (17)	0.589
Other (atrial myxoma, ascending aorta)	1 (14)	1 (4)	
Thoracic surgery, n (%)	0	1 (4)	
Cardiopulmonary bypass time (min, mean (SD)) (*n* = 29)	90 (46)	99 (44)	0.617
Respiratory parameters			
Tidal volume (ml kg^−1^ of predicted body weight, mean (SD),	7.8 (0.5)	7.8 (0.7)	0.957
Total PEEP (cmH_2_O, mean (SD))	5 (1)	5 (1)	0.443

### Effect of FC on haemodynamic parameters in the overall study population

Prior to FC, median E_A_ was 2.3 [1.7–2.8] mmHg.ml^− 1^, median E_LV_ was 1.5 [1–1.7] mmHg.ml^− 1^, and median E_A(ESP)_/E_LV_ ratio was 1.8 [1.3–2.3]. Twenty-three (80%) of the 30 patients were classified as “uncoupled”. After FC, median E_A_ was 2.1 [1.5–3], median E_A_/E_LV_ ratio was 1.6 [1.3–2.1], and median E_LV_ was 1.4 [0–1.7]. Twenty-three (77%) of the 30 patients were classified as fluid responders. Most uncoupled patients (21 out of 23 (91%); *p* = 0.003) were fluid responders.

### Effect of FC on haemodynamic parameters depending on SV changes

**Fig. 2:**
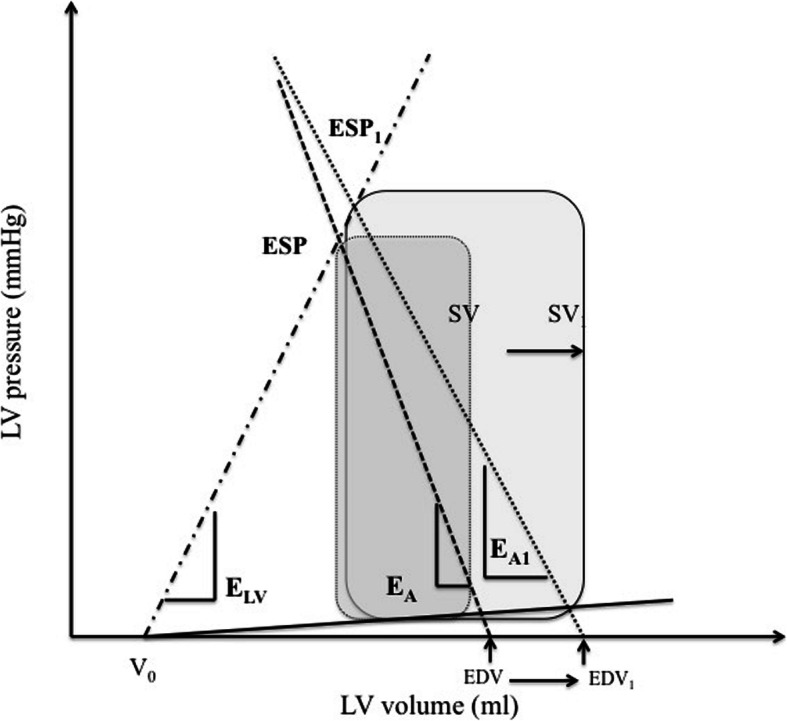
Changes in ventricular pressure-volume relationships in fluid responders with fluid challenge. Stroke volume increased (SV➔SV_1_), and end-diastolic volume (EDV➔EDV_1_) increased more than end-systolic pressure (ESP➔ESP_1_), resulting in decreased arterial elastance (E_A_➔E_A1_). Ventricular elastance (E_EL_) remained unchanged, resulting in a decreased E_A_/E_EL_ ratio

At baseline, E_A_, E_A_/E_LV_, were higher and C_A_, CI, SVi were lower in fluid responders than in fluid non-responders (Table 2, Fig. 2). In fluid responders, FC was associated with higher values for blood pressure, SVi, CI, SW, PVA, and SW/PVA ratio, and lower values for HR, SVRi, E_A_ and E_A_/E_LV_ ratio (Table 2, Fig. 2, supplementary file 1). In fluid non-responders, FC was associated with higher values for CVP and E_A_/E_LV_ ratio and a lower SW/PVA ratio (Table 2).

**Table 2:** Comparison of haemodynamic parameters in fluid responders and non-responders. Values are expressed as mean (SD) or median [interquartile range]. *CI* indexed cardiac output, *E*_*A*_ arterial elastance, *E*_*LV*_ ventricular elastance, *FC* fluid challenge, *HR* heart rate, *LVEF* left ventricular ejection fraction, *MAP* mean arterial pressure, *PVA* pressure volume area, *SAP* systolic arterial pressure, *SVi* indexed stroke volume, *SVRi* indexed systemic vascular resistance, *SW* stroke work; ^$^: *p* < 0.05 within groups (pre−/post-FC)

Haemodynamic variables	SV Non-responders (***n*** = 7)	SV Responders (***n*** = 23)	***p***-value
**HR (bpm)**			
Pre-FC	81 (23)	84 (22)	0.863
Post-FC	79 (19)	77 (18) ^$^	0.861
**SAP (mmHg)**			
Pre-FC	100 (21)	103 (16)	0.704
Post-FC	109 (22)	125 (22) ^$^	0.085
**MAP (mmHg)**			
Pre-FC	71 (12)	73 (12)	0.746
Post-FC	76 (12)	86 (14) ^$^	0.110
**LVEF (%)**			
Pre-FC	54 (11)	48 (11)	0.202
Post-FC	51 (8)	50 (10) ^$^	0.917
**SVi (ml m**^**−2**^**)**			
Pre-FC	28 (8)	22 (7)	0.050
Post-FC	25 (5)	29 (10) ^$^	0.401
**CI (ml min**^**−1**^**m**^**− 2**^**)**			
Pre-FC	2.3 (0.9)	1.7 (0.5)	0.045
Post-FC	1.9 (0.8)	2.2 (0.6) ^$^	0.487
**Arterial elastance (E**_**A**_**) (mmHg ml**^**−1**^**)**			
Pre-FC	1.8 [1.4–2.2]	2.5 [1.8–3.1]	0.033
Post-FC	2 [1.6–2.6]	2.2 [1.5–3.2] ^$^	0.774
**Arterial compliance (ml mmHg**^**−1**^**)**			
Pre-FC	1.6 (0.9)	0.94 (0.32)	0.014
Post-FC	1.1 (0.5)	0.98 (0.37)	0.455
**SVRi (mmHg ml**^**−1**^**m**^**−2**^**)**			
Pre-FC	34 (13)	47 (14)	0.055
Post-FC	37 (10)	38 (11) ^$^	0.672
**Ventricular elastance (E**_**LV**_**) (mmHg ml**^**−1**^**)**			
Pre-FC	1.6 (1.1–2)	1.5 (0.9–1.7)	0.564
Post-FC	1.5 (1.1–2.4)	1.5 (0.9–1.7)	0.564
**E**_**A**_**/E**_**LV**_**ratio**			
Pre-FC	1.2 (0.9–1.4)	1.9 (1.7–2.2)	0.007
Post-FC	1.4 (0.9–1.8) ^$^	1.5 (1.3–2) ^$^	0.144
**SW (joules)**			
Pre-FC	4769 (1513)	3672 (1349)	0.077
Post-FC	4691 (1690)	6032 (2040) ^$^	0.126
**PVA (joules)**			
Pre-FC	6919 (1705)	6111 (9132)	0.329
Post-FC	7319 (1964)	9164 (2868) ^$^	0.124
**SW/PVA ratio**			
Pre-FC	0.69 (0.13)	0.60 (0.14)	0.136
Post-FC	0.64 (0.13) ^$^	0.66 (0.12) ^$^	0.761

### Correlations between haemodynamic parameters, E_A_ and their changes

E_A_ changes were correlated with those of SV (*r* = 0.858, *p* < 0.001), SVRi (*r* = − 0.898, *p* < 0.001), C_A_ (*r* = 0.729, *p* < 0.001), and LVEF (*r* = − 0.620, *p* < 0.001). MAP changes were correlated with those of E_A_ (*r* = 0.479, *p* = 0.0001), SVRi (*r* = 0.468, *p* = 0.009), E_LV_ (*r* = 0.421, *p* = 0.021), and C_A_ (*r* = − 0.543, *p* = 0.002).

### Predictive value of E_A_, E_LV_, E_A_/E_LV_ ratio and PPV

At baseline, the E_A_/E_LV_ ratio predict fluid responsiveness with an AUC of 0.84 [95% confidence interval (95% CI)] (0.66–1), An E_A_/E_LV_ cut-off of 1.4 gave a sensitivity of 87% [66–97], a specificity of 86% [42–100], a positive likelihood ratio of 6.1, negative likelihood ratio of 0.15, a positive predictive value of 95 and a negative predictive value of 67. With an AUC [95% CI] of 0.75 [0.58–0.94] (*p* = 0.001), E_A_ was poorly predictive of fluid responsiveness. With an AUC [95% CI] of 0.39 [0.13–0.66] (*p* = 0.541), E_LV_ did not predict fluid responsiveness.

## Discussion

In fluid responders, V-A coupling was characterized by a high pre-challenge E_A_/E_LV_ ratio due to high E_A_ with respect to E_LV_. Because FC was associated with SV increase, it has improved V-A coupling. The baseline E_A_/E_LV_ ratio was a predictor of fluid responsiveness. This effect was associated with a decrease of E_A_ without changes of E_LV_. E_A_ changes were related to those of systemic vascular resistance (continue component) and of arterial compliance (pulsatile component). Improving V-A coupling was associated with a better myocardial work efficiency (i.e lower myocardial work for a better SV and blood pressure).

Few studies have specifically evaluated the effect of FC on V-A coupling. One study in postoperative cardiac surgical patient found an increase in SW, PVA and afterload, as a result of increased SV [21]. A subsequent study of septic shock patients assessed the impact of FC on E_A_ and its components [17]. According to V-A coupling concept, an increase of preload may lead to an increase of SV and E_A_ [2]. We observed a decrease after FC [21]. These results are close to those of Guarracino et al. who demonstrated a decrease of E_A_ with FC [17]. Several explanations for our findings can be proposed.

The sympathetic nervous system plays a key role (via the baroreflex) in regulating blood volume, blood flow and blood pressure [22]. Accordingly, preload-dependent patients probably have higher levels of sympathetic activation than non-dependent patients, as evidenced by higher E_A_, higher systemic vascular resistance, and lower arterial compliance values at a given blood pressure. This response is designed to adapt blood flow to the patient’s needs, which appears to be effective, as ScVO_2_ and arterial lactate levels were not significantly different between the two groups of patients. FC restores preload and blood flow, and thus meets the patient’s needs. A decrease in E_A_ might be caused by several interlinked mechanisms affecting the resistive component (HR and SVR) and the pulsatile component (C_A_) of arterial load. The increase in blood pressure induced by an increase in CO decreases sympathetic activation, systemic vascular resistance and E_A_. The baroreflex has been shown to maintain adequate blood pressure by modulating E_A_, E_LV_ and blood volume [23]. An increase in blood flow decreases vascular tone by activating the NO pathway and by initiating vascular recruitment [24]. As a result of shear stress, blood flow modulates the diameter of blood vessels and can influence aortic compliance [25]. Segers et al. used a heart-artery interaction model to show that the contribution of the resistive component to this effect is threefold higher than that of the pulsatile component [26].

### V-A coupling in clinical practice

The present study demonstrated that neither E_A_ nor E_LV_, but only E_A_/E_LV_ ratio defines the response of left ventricle to fluid expansion. In other words, the E_A_/E_LV_ ratio, which characterizes the interaction between the ventricle and the arterial vascular system defines which patient will be fluid responder. We specifically included patients not treated with vasopressors and inotropes to avoid any treatment-related bias. Hence, we can safely assume that our results were related to the sole effect of FC. Based on our results and those of previous studies performed in ICU, anaesthesia and cardiologic area, the V-A coupling represents a hemodynamic model that is able to predict the effects of each hemodynamic therapeutic, and their impact on clinical outcomes [6–9]. Authors have already demonstrated that optimizing V-A coupling was associated with better lactate clearance and patient survival [8, 9].

When the E_A_/E_LV_ ratio is over 1.36, the main determinant to be firstly optimized should be E_A_. Arterial elastance is easy to obtain at bedside. Hypotensive preload dependent patients have often V-A uncoupling because of high E_A_ (> 2.2) in relation to low SV and higher sympathetic activation in order to maintain tissue perfusion. The hemodynamic therapeutic would be to decrease E_A_ by using FC [17]. The second step may evaluate E_LV_ to evaluate the E_A_/E_LV_ ratio. One limitation could be the measure of E_LV_ because of Chen ‘s formula. A mobile phone application has been developed to easily calculate E_LV_. Simpler formulas have been developed but they are not interchangeable to track variations of E_A_/E_LV_ ratio with therapeutic intervention [27].

Another method based on V-A coupling could be the use of the dynamic arterial elastance which is the ratio of stroke volume variation to pulse pressure variation [28]. The dynamic arterial elastance can be easely measured with invasive and non-invasive hemodynamic devices [29–31]. Dynamic arterial elastance is a real time indicator of the interaction between the heart and the vascular system that has been challenged as an indicator of V-A coupling [32, 33]. Several clinical studies have demonstrated that dynamic arterial elastance is able to predict SV and blood pressure response to FC or norepinephrine, and it can be used to optimize treatment [17, 29–32, 34]. Recently, dynamic arterial elastance was demonstrated to be inversly correlated to V-A coupling, and track its changes [33]. The final clinical relevance of V-A coupling for ICU patients will require well designed interventional trials evaluating a hemodynamic optimization based on V-A coupling, its determinants or a surrogate such as dynamic arterial elastance.

This study presents a number of limitations. We did not register the study on clinical trial database. The study population may have differed from septic shock patients. Most of our patients presented perioperative hypovolaemia, whereas septic patients generally have acute circulatory failure with a combination of hypovolaemia, changes in microvascular perfusion and central-to-peripheral arterial decoupling. The methods used to calculate E_LV_ and E_A_ can be open to criticism because we did not use a high-fidelity ventricular pressure catheter. We measured E_LV_ by a non-invasive single-beat method based on a linear end-systolic pressure-volume relationship, and a constant volume axis intercept of the end-systolic pressure volume relationship [10, 29]. Calculation of E_LV_ assumes that the end-systolic pressure-volume relationship is load-independent, with a linear slope, and that V_0_ is not influenced by inotropes [29]. We calculated ESP from a radial artery signal, which may differ from the aortic pressure signal. However, radial artery pressure has been reported to provide a good estimate of ESP [14, 35]. It can be argued that estimation of ESP from the radial artery has not been fully validated, any error in this method would only affect the precision of absolute values of E_A_ and E_LV,_ but not the E_A_/E_LV_ ratio, as the error in end-systolic pressure would be similar. The predictive value of E_A_/E_LV_ for increased SV can therefore be considered to be valid. Arterial load assessment was based on a two-element Windkessel model and integrative simplification. More precise models have been developed, such as three- and four-element Windkessel models that include arterial impedance and wave reflection. However, these methods would be difficult to apply at the bedside. Despite these limitations, non-invasive evaluation of E_LV_ and E_A_ was validated against the gold standard method, and has been used in cardiac surgery [5]. In the present study, E_A_ and E_LV_ must be considered to be approximations of E_A_ and E_LV_.

## Conclusions

In fluid responders, V-A coupling was characterized by a high pre-challenge E_A_/E_LV_ ratio due to high E_A_ with respect to E_LV_. Because FC was associated with SV increase, it has improved V-A coupling. This effect was associated with a decrease of E_A_ without changes of E_LV_. Measuring V-A coupling can characterize the patient’s haemodynamic status and predict the cardiovascular system’s response to FC. Further studies are need to confirm the clinical relevance of optimizing V-A coupling in ICU patients.

## Supplementary information

**Additional file 1.**

## Data Availability

Unfortunately, we do not have permissions to share these data. However, we would be happy to collaborate with requests from individual research groups would like to access our raw data. Please contact the corresponding author for further information.

## Abbreviations

C_A_: Arterial compliance
CO: Cardiac output
CI: Cardiac index
CV: Coefficient of variation
CVP: Central venous pressure
E_A_: Arterial elastance
E_LV_: Ventricular elastance
EDV: End diastolic volume
ESP: End systolic pressure
ESV: End systolic volume
FC: Fluid challenge
HR: Heart rate
LVEF: Left ventricular ejection fraction
LSC: Least significant change
PVA: Pressure volume area
V-A coupling: Ventriculo-arterial coupling
SVRi: Indexed systemic vascular resistance
SV: Stroke volume
SW: Stroke work
