# Supplementary material for: Fluid expansion improve ventriculo-arterial coupling in preload-dependent patients: a prospective observational study
Source: BMC Anesthesiol. 2020 Jul 17;20:171. doi: 10.1186/s12871-020-01087-7 (PMC7366889; doi:10.1186/s12871-020-01087-7)
Supplement: Supplementary file 1 — Additional file 1. [file 12871_2020_1087_MOESM1_ESM.docx]

**Table 2. Comparison of haemodynamic parameters in fluid responders and non-responders.** Values are expressed as mean (SD) or median [interquartile range]. **CVP**, central venous pressure; **DAP**, diastolic arterial pressure; **E_A_,** arterial elastance; **E_LV_**, ventricular elastance, **FC**, fluid challenge; **HR**, heart rate; **LVEF**, left ventricular ejection fraction; **MAP**, mean arterial pressure; **SAP**, systolic arterial pressure; **^$^**: *p*<0.05 within groups (pre-/post-FC).

| **Haemodynamic variables** | **SV Non-responders**  **(n=7)** | **SV Responders**  **(n=23)** | ***p-value*** |
| --- | --- | --- | --- |
| **SAP (mmHg)**  Pre-FC  Post-FC | 100 (21)  109 (22) | 103 (16)  125 (22) ^$^ | 0.704  0.085 |
| **DAP (mmHg)**  Pre-FC  Post-FC | 58 (11)  60 (12) | 58 (11)  66 (13) ^$^ | 0.829  0.265 |
| **MAP (mmHg)**  Pre-FC  Post-FC | 71 (12)  76 (12) | 73 (12)  86 (14) ^$^ | 0.746  0.110 |
| **CVP (mmHg)**  Pre-FC  Post-FC | 6 (3)  8 (2) ^$^ | 6 (3)  8 (3) ^$^ | 0.775  0.981 |
| **ESV (ml)**  Pre-FC  Post-FC | 52 (21)  58 (23) | 58 (25)  60 (26) | 0.566  0.835 |
| **EDV (ml)**  Pre-FC  Post-FC | 101 (22)  95 (20) | 86 (33)  112 (42) ^$^ | 0.300  0.331 |
| **Arterial elastance (E_A_ (mmHg ml^-1^)**  Pre-FC  Post-FC | 1.8 [1.4-2.2]  2 [1.6-2.6] | 2.5 [1.8-3.1]  2.2 [1.5-3.2] ^$^ | 0.033  0.774 |
| **Ventricular elastance (E_LV_) (mmHg ml^-1^)**  Pre-FC  Post-FC | 1.6 (1.1-2)  1.5 (1.1-2.4) | 1.5 (0.9-1.7)  1.5 (0.9-1.7) | 0.564  0.564 |
| **Arterial lactate (mmol l^-1^)**  Pre-FC  Post-FC | 1.5 (1.2-3.4)  1.5 (1.4-2) | 1.4 (1.1-2.2)  1.3 (1.1-2.1) | 0.174  0.328 |
| **ScVO_2_**  Pre-FC  Post-FC | 65 (6)  60 (8) | 63 (11)  64 (8) | 0.824  0.399 |
